# FlexSketch: Estimation of Probability Density for Stationary and Non-Stationary Data Streams

**DOI:** 10.3390/s21041080

**Published:** 2021-02-04

**Authors:** Namuk Park, Songkuk Kim

**Affiliations:** School of Integrated Technology, Yonsei University, Incheon 21983, Korea; namuk.park@yonsei.ac.kr

**Keywords:** probability density estimation, streaming data, sensor system

## Abstract

Efficient and accurate estimation of the probability distribution of a data stream is an important problem in many sensor systems. It is especially challenging when the data stream is non-stationary, i.e., its probability distribution changes over time. Statistical models for non-stationary data streams demand agile adaptation for concept drift while tolerating temporal fluctuations. To this end, a statistical model needs to forget old data samples and to detect concept drift swiftly. In this paper, we propose FlexSketch, an online probability density estimation algorithm for data streams. Our algorithm uses an ensemble of histograms, each of which represents a different length of data history. FlexSketch updates each histogram for a new data sample and generates probability distribution by combining the ensemble of histograms while monitoring discrepancy between recent data and existing models periodically. When it detects concept drift, a new histogram is added to the ensemble and the oldest histogram is removed. This allows us to estimate the probability density function with high update speed and high accuracy using only limited memory. Experimental results demonstrate that our algorithm shows improved speed and accuracy compared to existing methods for both stationary and non-stationary data streams.

## 1. Introduction

Estimating the probability density function (PDF) of a random variable based on a stream of data samples from sensors is a fundamental problem arising in a broad range of areas such as machine learning [1], data structures [2], and systems [3]. There are two recent challenges in this problem.

First, the characteristics of a data stream often change. This might be due to the accidental fluctuation caused by an insufficient number of samples. In this case, a sufficient amount of data could reduce the fluctuation gradually. In some other cases, the data stream itself is non-stationary. In other words, the probability distribution of a random variable over the data stream, called concept, changes over time, which is called concept drift [4]. Concept drift occurs in many types of data such as temporal sensor data [5], video [6], and spatiotemporal data [7]. A static model constructed with the assumption of stationarity of the data stream may lead to an erroneous conclusion under the presence of concept drift. Therefore, there is a need for a method that can estimate PDFs adaptively according to concept drift for practical applications.

Second, real-world applications need to be able to handle increasing amounts of data and high-speed data streams while keeping low latency. Therefore, the demand for an online algorithm to estimate PDFs with high speed and high accuracy using only a small amount of memory is ever-increasing. While there exist online PDF estimation algorithms in literature [8,9,10,11], they usually cannot update probability models at high speed. Furthermore, they cannot adapt well to various types of data streams including those with concept drift.

**Figure 1:**
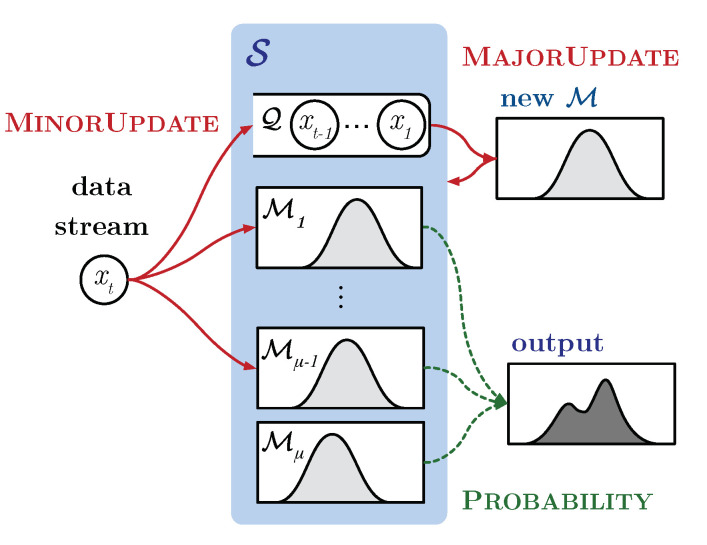
Overview of the proposed FlexSketch.

In order to deal with these challenges, we propose FlexSketch, which is an online probability density estimation algorithm that achieves high update speed and high accuracy with only a small amount of memory for both stationary and non-stationary data streams. As shown in Figure 1, FlexSketch estimates the PDF by using an ensemble structure composed of several statistical models. In particular, we exploit histogram for the statistical models, which allows fast and low-memory operations for data streams. Each histogram represents statistics of a different length of data history. FlexSketch updates each histogram for a new data sample and builds a new histogram when it detects the concept drift of input data. By decoupling updating each statistical model from amending the composition of the ensemble, FlexSketch achieves high accuracy both for stationary and non-stationary data streams.

To adapt non-stationary data streams in an efficient way, a single operation updating the estimated PDF according to the data stream is divided into two elementary operations: “fast and minor update operation” (MinorUpdate) and “slow and major update operation” (MajorUpdate). The type of operation to be performed varies depending on the characteristics of the data stream. When minor changes occur in a data stream, FlexSketch updates the PDF for the data stream at high speed by using MinorUpdate. This operation simply updates each model in FlexSketch. In contrast, when major changes occur in the data stream, FlexSketch updates the PDF by using MajorUpdate, which builds a new model including the recent data stream and adds it to FlexSketch. Finally, FlexSketch has multiple versions of a model, ranging from a version representing only recent data to a version representing both recent and old data. FlexSketch constructs the PDF by linear combination of these coarse models.

FlexSketch dynamically decides when to forget old data and to build a new statistical model by measuring divergence between the current model and recently sampled data. This allows FlexSketch to stay stable when the concept of the data stream does not change, and to tolerate temporal out-linear data. In addition, FlexSketch achieves agile adaptation to sudden or incremental concept drift since MajorUpdate integrates a newly built histogram, which amends the statistical model. Though the histogram is a compact data structure and easy to maintain, it may provide coarse information about probability distribution. FlexSketch alleviates this problem by incorporating an ensemble of histograms.

The current implementation of FlexSketch only supports one-dimensional data. There are many applications relying on statistical modeling of one-dimensional data streams such as online anomaly detection [5,12], fault-detection [13] and DDoS detection [14], which are potential areas where FlexSketch is applicable. While many online sensor-based applications handle one-dimensional data, other applications need to deal with multi-dimensional data streams. Since the limited dimension of FlexSketch circumscribes the application of FlexSketch in some areas such as machine learning for high dimensional data [15] and anomaly detection based on multivariate data [16], FlexSketch needs to be expanded to handle multi-dimensional data for wider applications.

The experimental results demonstrate that FlexSketch updates PDFs for data streams up to 16× faster than the alternatives while using only a limited amount of memory. Moreover, FlexSketch adapts well to various types of concept drift, and it is more accurate than the alternatives.

The main contributions of FlexSketch are as follows:

- We propose a new method to estimate probability distribution for data streams with concept drift.
- FlexSketch decouples adapting to concept drift from adjusting the statistical model for stationary data by incorporating two separate operations.
- FlexSketch achieves low computational overhead and high throughput, which are critical for processing of stream data, using an ensemble of compact histograms.

The remainder of the paper is organized as follows. Section 2 briefly surveys the related work. In Section 3, the proposed FlexSketch is described in detail. Section 4 presents extensive experimental results. Finally, the conclusions are given in Section 5.

## 2. Related Work

There are multiple research topics to deal with data streams with concept drift [17]. Supervised classification of data streams with concept drift is also studied extensively, e.g., [18,19,20,21]. The core parts of these researches are how to detect concept drift, how to forget old data, and how to rebuild a new statistical model. Some methods [9,22] gradually update statistical model without explicit detection of concept drift. Other studies attempt to detect concept drift in batch-based methods [23,24,25,26] and online methods [27,28,29]. While [30,31] rely on process control, FlexSketch detects concept drift using a multiple-window-based method like [32]. Researches also focused on how to measure difference in distribution between recent data and old data. The common methods are based on entropy or KL-divergence [33,34,35]. We introduce an error based metric to detect concept drift in Section 3.1.

To deal with non-stationary data, statistical models should be able to forget old data or to depreciate their contribution. Some methods [9,36,37] decay the importance of old data linearly or exponentially. This approach is good for tolerating temporal fluctuations. Since gradual decaying is slow to adapt to sudden concept drift, [38,39] use sliding window mechanisms to keep some recent data and to discard old data. FlexSketch deploys both gradual decaying and abrupt forgetting. When the input data is stationary, FlexSketch depreciates old data exponentially. However, when FlexSketch detects concept drift, it discards the oldest histogram and incorporates a new histogram, which allows FlexSketch agile adaptation.

There exist many kinds of density estimation algorithms for data streams. Traditionally, kernel density estimation is performed as a batch-processing algorithm for density estimation of a dataset. There are some algorithms [9,10,11] generalized to online processing for adaptive density estimation. They usually constitute a Gaussian mixture model by assigning a Gaussian kernel to newly added data and thereafter merge kernels based on certain rules. Particularly, the online kernel density estimation (denoted by oKDE in this paper) [9] can adapt to concept drift by enabling to forget past data. However, this method is slow in updating the estimated probability density due to the requirement of relatively massive calculation. In contrast, we focus on developing an efficient mechanism that can update the probability density adaptively by exploiting updating operations having different levels of computational complexity.

There also exist methods to estimate the distribution of a data stream based on histograms, including the streaming parallel decision tree (denoted by SPDT in this paper) [8], variations of the V-optimal histogram algorithm [40,41] and quantile summarization algorithms [42,43,44,45]. However, they cannot forget the contribution of the past data and adapt to various types of concept drift. To solve this problem, model maintenance strategies using fixed and variable size sliding windows [46,47,48] for histograms can be used. [7] proposes a histogram-based sketch mechanism with gradual forgetting. However, it is unknown whether they guarantee satisfactory performance when different types of concept drift occurs. On the other hand, FlexSketch efficiently updates the statistical model for both high accuracy and high efficiency by amending the composition of the ensemble.

There are some studies using ensemble methods, e.g., [49,50,51]. While some methods [33,34] use hierarchical structure, FlexSketch uses flat combination of compact and simple data structure like [19,52]. Most of previous works focus on improving accuracy of supervised classification for data streams with concept drift. On the other hand, FlexSketch uses the ensemble technique to improve the update speed of density estimation.

## 3. Proposed Method

The goal of our method is to estimate the PDF of stationary (i.e., without concept drift) and non-stationary (i.e., with concept drift) data stream at high speed and high accuracy while using a small amount of memory. Here, the meaning of accurately estimating the PDF for a stationary data stream is straightforward. On the other hand, for a non-stationary case, it is not simple and has several aspects. We consider the accuracy of density estimation for a non-stationary data stream from three points of view. First, the estimated PDF should forget old concepts quickly after concept drift occurs. Second, the estimated PDF should adapt to the latest concept as soon as a concept drift occurs. Third, the estimated PDF should remain stable even if an accidental outlier occurs in the data stream.

To fulfil these requirements, the proposed method is built on the following ideas.

(a) We choose a histogram as the statistical model. When there are only minor changes in the data stream, a histogram is a suitable model since it can be updated at a high speed.
(b) Our method uses an ensemble data structure consisting of several histograms. The ensemble structure can compensate for inaccuracy of a histogram.
(c) We design two adaptation techniques. If the data stream is stationary or there are only minor changes in it, FlexSketch updates the models, i.e., histograms. On the other hand, if there are major changes in the data stream, updating the models may not guarantee sufficient accuracy. To address this issue, we generate a new model that represents the changed data stream and adds it to the data structure.

Let S denote the data structure of the FlexSketch framework. S consists of a recent data stream and multiple versions of a statistical model as follows:

(1) $$S=\{Q,M_{1},\cdots ,M_{N}M,n_{1},\cdots ,n_{N}M\}$$

where Q is the buffer for the recent dataset given through the input data stream, $M_{i}$ is the *i*th histogram, $N_{M}\geq 2$ is the total number of histograms, and $n_{i}$ is the number of data used to update $M_{i}$. Since we build a new histogram when a major change in the data stream is detected, the histograms in S are created at different times. As a convention, $M_{1}$ is the most recently added one and $M_{N_{M}}$ is the oldest one. This means that $M_{i+1}$ is older than $M_{i}$ and thus undergoes more updates. Therefore, $n_{i+1}>n_{i}$. Each histogram is a set of disjoint intervals called bins ($I_{j}$) and the frequency count ($m_{j}$) for each bin, where $j=1,\ldots ,N_{B}$:

(2) $$M=\{I_{1},\cdots ,I_{N_{B}},m_{1},\cdots ,m_{N_{B}}\}.$$

Two important operations of FlexSketch are (a) the update operation for the data stream online and (b) the query operation to obtain the probability for a certain data, which are explained below.

### 3.1. Update Operation

Algorithm 1 summarizes the operation to update S with a given data sub-stream *X* whose number of data is |X|. First, S is updated at a minor level using operation MinorUpdate and *X* is appended in the buffer. If the size of the buffer *Q* exceeds a threshold $N_{Q}$, i.e., if only the minor update has been performed with a certain number of data, the adequacy of the most recently added model $M_{1}$ is examined by operation Diagnose. If a large discrepancy is found from the operation between the recent data stream stored in *Q* and $M_{1}$, the major update operation (MajorUpdate) is performed and the buffer is cleared.
**Algorithm 1:** Update operation.
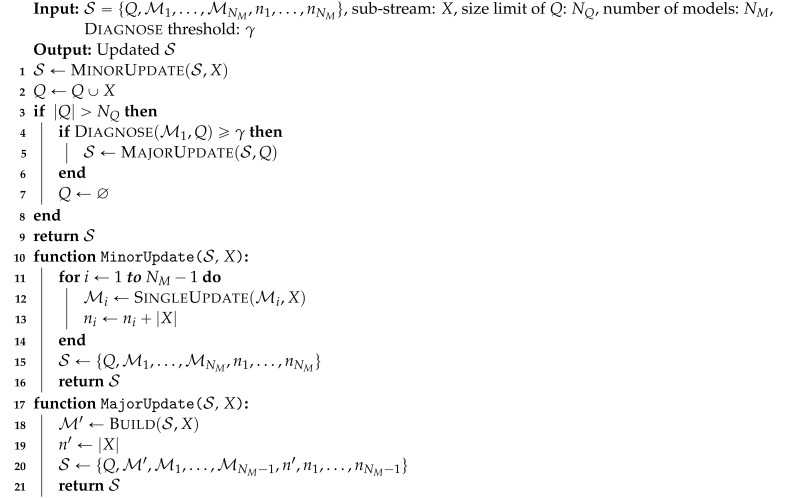


The MinorUpdate operation causes only minor changes of S. The steps of the operation are shown in lines 10 to 16 of Algorithm 1. Each histogram in S except the oldest one ($M_{N_{M}}$) is updated by operation SingleUpdate. This operation consists of two steps. First, it searches the bin $I_{j}$ whose interval contains each data in *X*, *x*. For fast search, we use the red-black tree method. Second, it increases the count of $I_{j}$, $m_{j}$, by 1. If there is no bin for *x*, *x* is ignored. This causes a discrepancy between the histogram and *X*, which is resolved by the MajorUpdate operation.

The Diagnose operation measures the amount of discrepancy between the most recent model $M_{1}$ and the data stream in *Q*. If the output of the operation is larger than a threshold γ, we consider that concept drift occurs and the MajorUpdate operation needs to be performed.

There are some requirements for the Diagnose operation. First, its result should be invariant under scaling transformation of the data, so that the threshold is independent of the scale of the data. Second, the result of Diagnose should be stable even when only a small number of data are given; otherwise, MajorUpdate is performed too frequently, which results in increased computational complexity, and the PDF estimation becomes inaccurate. Third, the result of Diagnose must be finite even if the input dataset is not included in the domain of the histogram; otherwise, the result will diverge whenever accidental outliers deviate from the domain.

**Figure 2:**
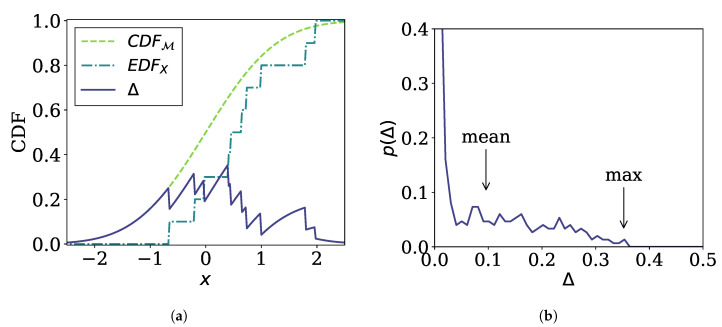
Example of the error function. (**a**) The error function is given as the difference (solid line) between the cumulative distribution function (CDF) of a model (dashed line) and the empirical distribution function (EDF) of a sub-stream (dash-dot line). (**b**) The probability density function (PDF) for the value of the error function.

To design a Diagnose operation satisfying these requirements, we first define the error function Δ(x) between *X* and M as the absolute difference between the cumulative distribution function (CDF) of M, $CDF_{M}\left(x\right)$, and the empirical distribution function (EDF) for *X*, $EDF_{X}\left(x\right)$ (Figure 2a):

(3) $$\Delta \left(x\right)=|CDF_{M}\left(x\right)-EDF_{X}\left(x\right)|.$$

Then, a representative value of the error function serves as the output of the Diagnose operation. We consider two options, i.e., the maximum value given by

(4) $$\overset{ˇ}{\varepsilon }=\max_{x}\Delta \left(x\right)$$

and the mean value given by

(5) $$\overset{ˉ}{\varepsilon }=\int_{0}^{1}\Delta \cdot p\left(\Delta \right)d\Delta$$

where p(Δ) is the PDF for Δ. $\overset{ˇ}{\varepsilon }$ or $\overset{ˉ}{\varepsilon }$ may be used as the result of Diagnose directly. Note that since Δ(x) is the difference between two probability distributions, its value can take only between 0 and 1, which is the range of the integration. However, we note that the range of $\overset{ˇ}{\varepsilon }$ and $\overset{ˉ}{\varepsilon }$, which is between 0 and 1, is too narrow for practical use. Thus, for convenience, we scale them to obtain the final output of Diagnose as follows:

(6) $$\delta =\frac{\varepsilon }{1-\varepsilon }$$

Note that δ=0 for ε=0, δ≃ε for small ε (i.e., ε≪1), and δ=∞ for ε=1. Therefore, the output of Diagnose ranges from 0 to ∞ through this scaling.

The MajorUpdate operation is shown in lines 18 to 21 in Algorithm 1. It first builds a new histogram $M^{\prime }$ with *X* through operation Build in order to accommodate a big change (i.e., concept drift) in the data stream. Then, the new histogram is enqueued to S as the first model of the ensemble and the oldest one ($M_{N_{M}}$) is dequeued from S.

The Build operation is shown in Algorithm 2. It basically creates a new histogram that covers the data ranges of both the existing histograms in S and the recent data, so that the new histogram can account for the characteristics of the recent data. First, the boundaries of the bins of the new histogram are obtained so as to uniformly split the range of the combined CDF (CDF of S and EDF of *X*) (lines 2 to 4 of Algorithm 2). Then, the EDF of *X* is used to obtain the count in each bin (lines 5 to 8 of Algorithm 2).
**Algorithm 2:** Build operation.
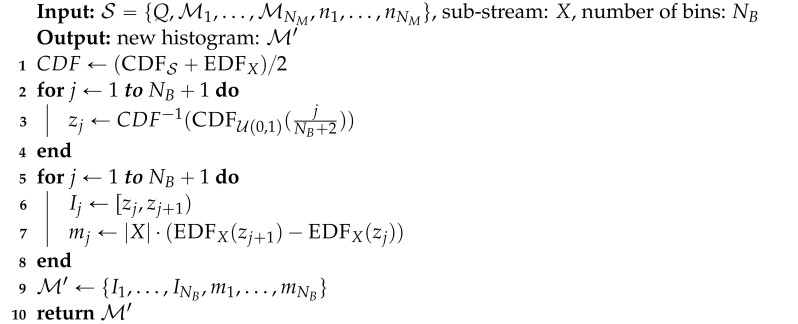


### 3.2. Query Operation

The query operation is to obtain the probability of a certain input data using S that has been established using the past data through the update operations explained above. As mentioned before, we employ an ensemble approach for this using the histograms contained in S. In other words, the probability of a given data *x* is calculated from S as a linear combination of the PDFs represented by the histograms $M_{i}$, i.e.,

(7) $$p_{S}\left(x\right)=\sum_{i=1}^{N_{M}}\alpha_{i}\cdot p_{M_{i}}\left(x\right),$$

where $p_{M_{i}}\left(x\right)$ is the probability of *x* from histogram $M_{i}$ and $\alpha_{i}$ is the weight of $M_{i}$. The former is given by the proportion of the data count for the bin to which *x* belongs, i.e.,

(8) $$p_{M}\left(x\right)=\left\{\begin{array}{cc} \frac{m_{j}}{|I_{j}|\cdot \sum_{k=1}^{N_{B}}m_{k}}, & x\in I_{j},\\ 0, & \mathrm{otherwise}, \end{array}\right.$$

where $|I_{j}|$ is the size of an interval and we omit the subscript *i* for simplicity. The weight $\alpha_{i}$ is determined in a way that a newer histogram receives a higher weight. Then, the final probability (7) depends more on the histograms that have been built more recently. For this, we use that an older histogram has been updated with more data, i.e., $n_{i+1}>n_{i}$. Specifically, the weight $\alpha_{i}$ is set to be negatively proportional to $n_{i}$, where the exponential function is used to ensure the weight value remains positive and, at the same time, to forget an old concept exponentially:

(9) $$\alpha_{i}=\frac{\exp(-n_{i-1}\lambda /N_{Q})}{\sum_{k=1}^{N_{M}}\exp\left(-n_{k-1}\lambda /N_{Q}\right)},$$

with $n_{0}=0$. Here, λ is a hyperparameter. Note that $\sum_{i=1}^{N_{M}}\alpha_{i}=1$ due to the normalization and $1>\alpha_{i}>\alpha_{i+1}>0$ because $n_{i}<n_{i+1}$.

To see how this works, let us consider the situation where concept drift occurs continuously so that we can assume that $n=n_{i+1}-n_{i}$ and $\alpha =\alpha_{i+1}/\alpha_{i}=\exp\left(-n\lambda /N_{Q}\right)$ for all *i*. And, let a PDF of concept $C_{a}$ changing over time be $p_{C_{a}}$ for positive integer *a* with $C_{1}$ being the latest one. Then, $p_{M_{1}}=p_{C_{1}}$, $p_{M_{2}}=p_{C_{1}}+p_{C_{2}}$, …, $p_{M_{N}M}=p_{C_{2}}+\cdots$ since MinorUpdate does not update the oldest model. Then, $p_{S}\propto \left(1+\alpha +\cdots +\alpha^{N_{M}-1}\right)p_{C_{1}}+\left(\alpha +\cdots +\alpha^{N_{M}}\right)p_{C_{2}}+\cdots$ holds. In other words, the contribution of the concept decreases at a rate of α. This means that FlexSketch forgets an old concept exponentially.

## 4. Experiments

We evaluate the computation time, accuracy, and memory usage of the proposed method for various types of stationary and non-stationary data streams. In particular, we compare our method with the two representative existing density estimation algorithms, oKDE [9] and SPDT [8].

### 4.1. Datasets

Stationary datasets: We consider three distributions. The first is a standard normal distribution, N(0,1), which appears frequently. The second is a bimodal distribution, 12N(−2,1)+12N(2,1), which is used to test if a density estimation algorithm can recognize multiple modes. The third is a log-normal distribution, lnN(0,1), which is used to test if an algorithm can estimate a long-tailed distribution. For all cases, one million data are randomly generated to follow the distributions.

Non-stationary datasets: For non-stationary datasets, we consider three types of concept drift as follows. For each case, we use one million data randomly sampled from the distribution.

**Figure 3:**
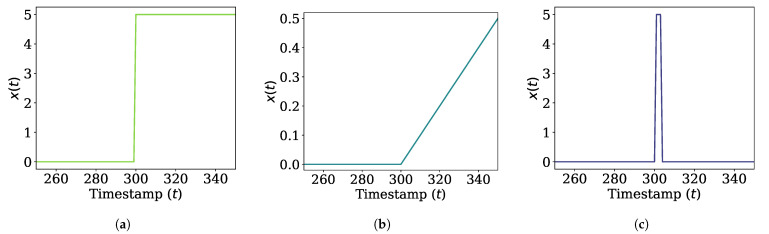
Changes in mean of distributions for data streams where concept drift occurs. (**a**) Sudden concept drift. (**b**) Incremental concept drift. (**c**) Blip concept drift.

(a) Sudden concept drift is defined as the case where the distribution of the data stream changes suddenly. It is to test how well a density estimation algorithm forgets old concepts after concept drift occurs. The underlying distribution is a normal distribution whose mean value changes abruptly, i.e., $N\left(x(t),1\right)$, where x(t)=0 for $t<t_{1}$ and $x\left(t\right)=x_{1}$ for $t\geq t_{1}$. We consider $t_{1}=300$ and $x_{1}=5$ as shown in Figure 3a.
(b) Incremental concept drift is defined as the case where the distribution of the data stream changes incrementally. It is to test how well a density estimation algorithm adapts to the latest concept. The underlying distribution is a normal distribution whose mean value moves at a constant speed, i.e., $N\left(x(t),1\right)$, where x(t)=0 for $t\leq t_{1}$ and $x\left(t\right)=v_{1}\cdot \left(t-t_{1}\right)$ for $t>t_{1}$. We set $t_{1}=300$ and $v_{1}=0.01$, as shown in Figure 3b.
(c) Blip concept drift is defined as the case where the distribution of data stream suddenly changes and returns to the original state in a short time. It is to test how well the estimated PDF remains stable even if an outlier occurs. The underlying distribution is a normal distribution whose mean value changes suddenly and returns, i.e., $N\left(x(t),1\right)$, where $x\left(t\right)=x_{1}$ for $t_{1}<t\leq t_{1}+t_{\epsilon }$ and x(t)=0 otherwise. $t_{\epsilon }$ is the duration of blip concept drift, which is set to $t_{\epsilon }=3$. We also set $x_{1}=5$ and $t_{1}=300$, as shown in Figure 3c.

### 4.2. Implementation

The parameters of FlexSketch are selected such that it has similar accuracy to oKDE and SPDT for the stationary data streams as follows: $N_{M}=3$, $N_{Q}=30$, λ=2.5, and γ=0.4. FlexSketch is implemented in Scala, which is publicly available at GitHub [53]. For oKDE, we use the JAVA implementation available in [54]. For SPDT, we use the Scala implementation in [55]. Note that the accuracy of SPDT decreases when concept drift occurs since SPDT stores the entire frequencies of the data stream. To address this issue, we modify SPDT by using a sliding window, which is referred to as SPDTw. The window size is set to 100 (this value was tuned such that SPDTw would exhibit similar accuracy to SPDT for stationary data. Increases in window size favor accuracy of stationary (or slowly changing) data streams to sudden concept drift, which reductions cause the inverse. Therefore, we calibrated these comparative methods for equitable results), for which SPDTw shows similar accuracy to FlexSketch for non-stationary data streams.

We perform all experiments on a machine with 4-core Intel CPU i7-7700K @ 4.2 GHz and 16 GB memory. The experiments run on a single thread. The version of Scala is 2.12.5 and the version of Java is 1.8.0.

### 4.3. Performance Metrics

Throughput We evaluate the computation times of the update and query operations of FlexSketch in million operations per second (Mops), which indicates the number of times per second our benchmark operation can be executed. There is a performance degradation in JVM in the first few iterations. Thus, we start to record the throughput after 20 iterations to warm up. Then, we record the mean value of the throughputs for the subsequent 30 iterations to minimize accidental deviations.

Error We measure the discrepancy between the estimated PDF and the ground truth distribution. We adopt the scaled mean average error (scaled MAE) of CDF, which is defined in (5) with scaling in (6), i.e., $\delta =\overset{ˉ}{\varepsilon }/\left(1-\overset{ˉ}{\varepsilon }\right)$.

Adaptability When concept drift occurs, the PDF estimated by a density estimation algorithm changes over time, so does the error. Thus, the mean of the error for a given time interval is not a sufficient metric for the accuracy of the algorithm for non-stationary data streams. Instead, we measure the adaptability of the algorithm using how the error changes over time. For this, we introduce a damped harmonic oscillator model in classical mechanics (e.g., a vibrating mass connected to a spring under damping) to represent the change in the error of the density estimation. In other words, the stability against outliers is equivalent to the resistance force (or frictional force) and the Update operation is equivalent to the restoring force. The density estimation algorithm tries to make the error smaller as the error increases and to keep the error unchanged as the error suddenly increases. Then, the governing equation for the time-dependent error δ(t) can be written as:

(10) δ¨(t)+δ¨0(t)+c·δ˙(t)+δ˙0(t)⏟resistance force+(k·δ(t)⏟restoring force=0

where $\delta_{0}\left(t\right)$ is the error between before and after the data distribution changes, $\dot{\delta }$ and $\ddot{\delta }$ are the first- and second-order time derivatives of δ, respectively, and *k* and *c* are model coefficients. *k* and *c* are determined by fitting the observed values of δ(t) and $\delta_{0}\left(t\right)$ to the model (10) under the assumption of over-damped oscillation (i.e., ${\left(c/2\right)}^{2}>k$). The solution is given by

(11) $$\delta \left(t\right)=\underset{\text{short-lived}\;\mathrm{term}}{\underbrace{A_{1}e^{-(c/2+\sqrt{c^{2}/4-k})t}}}+\underset{\text{long-lived}\;\mathrm{term}}{\underbrace{A_{2}e^{-(c/2-\sqrt{c^{2}/4-k})t}}}$$

where $A_{1}$ and $A_{2}$ are constants. Based on the fitted model, the following four performance metrics are derived.

(a) Half-life: In order to measure the adaptability of an algorithm under sudden concept drift, we measure the time taken until the error at the time of concept drift is reduced by a half, which is denoted as half-life:
  (12) t12=δ−112·δ(t1).
  This metric basically measures how quickly an old concept is forgotten in the short term.
(b) Lifetime: Similarly, we also quantify how long the contribution of the past data stays, or equivalently, how quickly the old concept is forgotten in the long term. The lifetime is defined as the time required for a long-lived term in (11) to reduce to 1/e times its initial value, which is given by
  (13) $$\tau ={\left(c/2-\sqrt{c^{2}/4-k}\right)}^{-1}.$$
(c) Lag: The lag measures how well the estimated PDF adapts to the data stream under incremental concept drift. It is defined as the absolute ratio of δ and the derivative of $\delta_{0}$ at t→∞, which can be obtained by
  (14) $$\left|\frac{\delta (\infty )}{{\dot{\delta }}_{0}\left(\infty \right)}\right|=\frac{c}{k}.$$
  If an algorithm does not adapt well to the concept drift, the accumulated error makes the algorithm lag behind more and more.
(d) Instability The instability measures how fast the estimated PDF moves for a short duration when blip concept drift occurs. It is defined as the velocity of the error, which can be approximated as
  (15) $$\sigma =\dot{\delta }\left(t_{1}\right)≃\frac{\delta \left(t_{1}+t_{\epsilon }\right)-\delta \left(t_{1}\right)}{t_{\epsilon }}.$$

Memory Usage The PDF estimated using the density estimation algorithm continues to use memory. After the estimation, this result or its changing history is recorded in the disk if necessary. Therefore, we record only the memory usage of the estimated PDF, but not the whole memory usage consumed by the Update or Query operation.

### 4.4. Throughput

We compare the throughput performance of the existing and our methods for the two key operations, i.e., updating the estimated density and producing the probability for a given data, which correspond to the Update and Query operations in our method, respectively.

UpdateFigure 4a shows the throughputs of the update operation of different density estimation algorithms for different types of data streams. The throughput of FlexSketch is 1.1 Mops, which is 16×, 16×, and 1800× higher than that of oKDE, SPDT, and SPDTw, respectively, for the stationary data streams. For the non-stationary data streams, the throughput of FlexSketch is 0.37 Mops, which is 5.8×, 5.7×, and 570× higher than that of oKDE, SPDT, and SPDTw, respectively. For the mixture data streams, the throughput of FlexSketch is 0.61 Mops, which is 9.7×, 9.3×, and 1000× higher than that of oKDE, SPDT, and SPDTw, respectively. We also perform the one-sample Wilcoxon signed-rank test under the hypothesis that the median of the throughput differences between the proposed method and the existing methods is zero, which confirms the significance of the differences (p<0.005). This result demonstrates that the additional computation time to manage multiple models is significantly smaller than the computation time to represent the data stream elaborately. This effect becomes more prominent when major concept drift does not occur. However, it shows a noticeable improvement even for a data stream with frequent major concept drift.

QueryFigure 4b shows the throughputs of different density estimation algorithms for the query operation. The throughput of FlexSketch is 0.47 Mops, which is similar to that of SPDT and SPDTw and smaller than that of oKDE. As shown in (7), FlexSketch linearly combines multiple models for each query. In order to improve the querying speed, we can add a caching algorithm, although it consumes 20–30% more memory. The throughput of FlexSketch is significantly improved with cache up to 4.1 Mops, which is 1.2×, 9.2×, and 9.4× higher than that of oKDE, SPDT, and SPDTw, respectively.

### 4.5. Accuracy (Error and Adaptability)

We compare the accuracy of FlexSketch with that of the alternatives by measuring errors for the stationary and non-stationary data streams.

#### 4.5.1. Stationary Case

**Figure 5:**
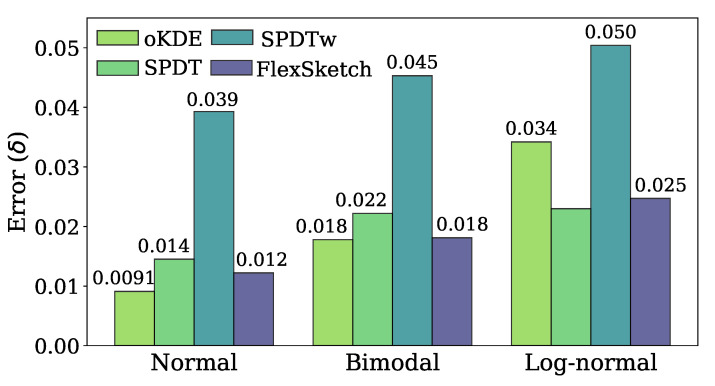
Estimation error (δ) for stationary data streams (a smaller value is better).

Figure 5 compares the estimation error of each algorithm after performing the update operation for three different stationary data streams. The error of FlexSketch for the normal distribution is 0.012, which is 0.75×, 1.2×, and 3.2× less than those of oKDE, SPDT, and SPDTw, respectively. It is intuitive that oKDE records the lowest error because it estimates the distribution by using a mixture of Gaussian distributions. SPDTw is less accurate than FlexSketch since the number of data used for update by SPDTw is limited to a fixed size within its window (note that the parameters of SPDTw are deliberately selected so as to have similar accuracy as FlexSketch when concept drift occurs, as mentioned in Section 4.2).

The error of FlexSketch for the bimodal distribution is 0.018, which is 0.98×, 1.2×, and 2.5× smaller than those of oKDE, SPDT, and SPDTw, respectively. oKDE using a Gaussian kernel shows the best result, as in the case of the normal distribution. And, the performance of FlexSketch is equal to that of oKDE within a margin of error. This indicates that the Build operation successfully constructs a new model that recognizes different modes well. Again, SPDTw is less accurate than FlexSketch for the aforementioned reason.

The error of FlexSketch for the log-normal distribution is 0.025, which is 1.4×, 0.93×, and 2.0× smaller than those of oKDE, SPDT, and SPDTw, respectively. Contrary to the results for the normal and bimodal distributions, FlexSketch and SPDT, which have high degrees of freedom, show smaller errors than oKDE.

#### 4.5.2. Non-Stationary Case

**Figure 6:**
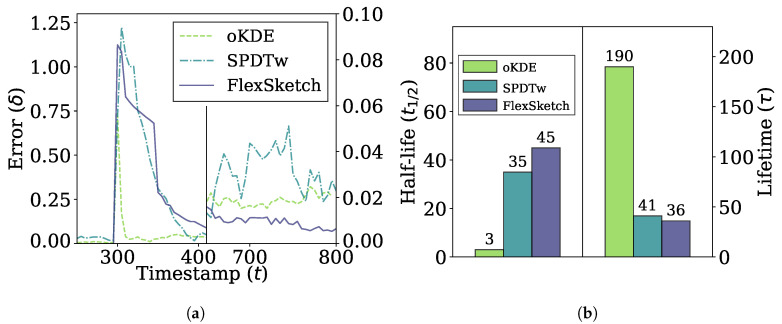
Error and adaptability performance under sudden concept drift. (**a**) Change of the error over time. The period between about 400 and 650 is omitted for better visualization, and the error after t = 650 is magnified with the scale at the right side. (**b**) Half-life (left side) and lifetime (right side). A smaller value is better.

Figure 6 shows the error and adaptability performance of different methods under sudden concept drift, i.e., the errors over time between the PDFs estimated using different algorithms and the underlying distribution of the data stream in Figure 6a, and the half-life and lifetime in Figure 6b. When the concept drift occurs at t=300, the errors jump to 1.0 or higher for all methods. As soon as the PDFs adapts to the new concept, the PDF forgets the old concept and the errors slowly fall to zero. In the short term, the error of oKDE decreases more quickly compared to SPDTw and FlexSketch, resulting in the shortest half-life by oKDE. However, oKDE shows the longest lifetime, indicating that it is affected by the old concept for a long time. In the long term, FlexSketch shows the smallest error in Figure 6a and also the shortest lifetime in Figure 6b.

We also measure the accuracy of the three methods over data generated by the MOA framework [56] for sudden concept change. Function 2 and 3 of the SEA generator [57] is used with a narrow drift-window (100 samples) to produce data streams and the error is measured against the mean of each cluster at the 2000 sample point after the drift. FlexSketch, SPDTw and oKDE exhibit errors of 0.27, 0.21 and 0.57, respectively. Though the overall trend is similar, FlexSketch shows a slightly higher error than SPDTw since the window size of SPDTw is small enough to evade from the effect of old data.

**Figure 7:**
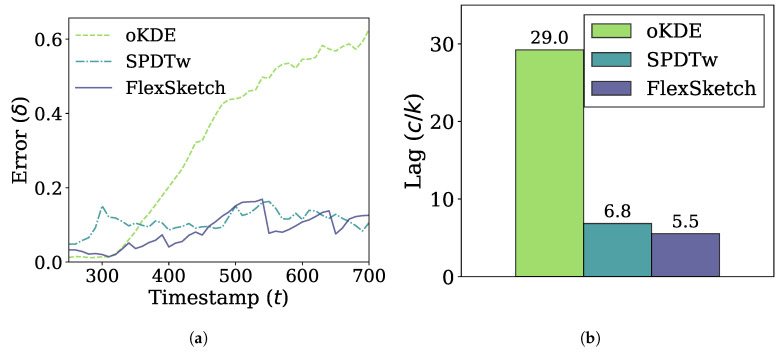
Error and adaptability performance under incremental concept drift. (**a**) Change of the error over time. (**b**) Lag (a smaller value is better).

In Figure 7, the performance of different methods under incremental drift is shown. The error of FlexSketch is saturated at 0.11, while those of SPDTw and oKDE are saturated at 0.14 and 0.58, respectively, as shown in Figure 7a. In addition, Figure 7b shows that FlexSketch has the smallest lag (5.3× and 1.2× smaller than those of oKDE and SPDTw, respectively). This demonstrates that FlexSketch can not only speed up computation, but also adapt more accurately to the changes in the data stream. This is also consistent with the observation for sudden concept drift that FlexSketch forgets the past concept faster than oKDE in the long term.

**Figure 8:**
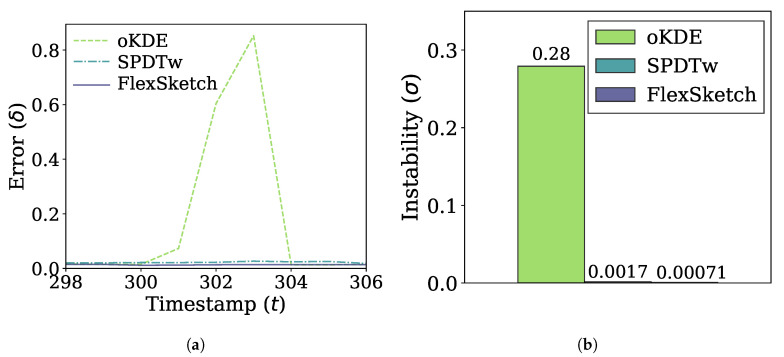
Error and adaptability performance under blip concept drift. (**a**) Change of the error over time. (**b**) Instability (a smaller value is better).

Figure 8a shows the errors of the three algorithms for the case of blip concept drift. The errors of FlexSketch and SPDTw increase only by 0.0021 and 0.0050, respectively, due to the blip concept drift, whereas oKDE shows a much larger increase of the error (up to 0.84). The comparison of the instability metric in Figure 8b also confirms that FlexSketch shows significantly improved performance, especially compared to oKDE.

### 4.6. Memory Usage

**Figure 9:**
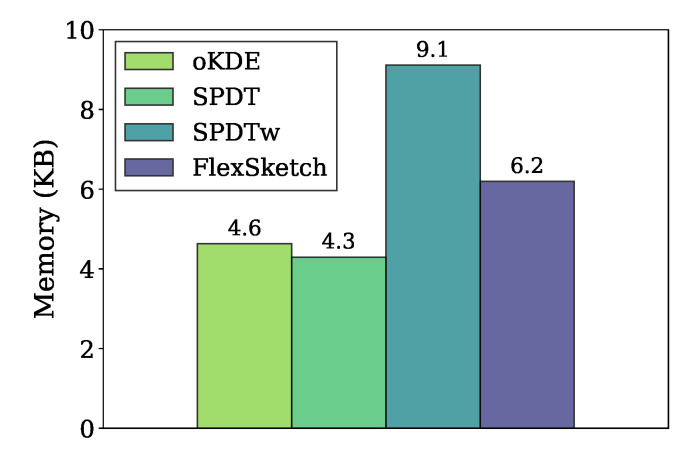
Comparison of memory usage.

Figure 9 compares the amount of memory used in the data structures of different density estimation algorithms for the stationary data stream. Our FlexSketch consumes 6.2 kbytes of memory, which is 1.3×, 1.4× and 0.68× more than that of oKDE, SPDT, and SPDTw, respectively. Since we set $N_{M}=3$, one could expect that FlexSketch requires 3× more memory usage than the others. However, the increased amount of memory consumption is much less than such an expectation by using efficient histogram computation.

### 4.7. Effects of Parameters

We investigate the effects of the algorithm parameters, i.e., $N_{M}$, $N_{Q}$, λ, and γ, on the performance in terms of throughput of the Update operation, error, and memory usage for the stationary data stream and the non-stationary data stream with incremental concept drift. The ranges of the parameters are as follows: 2 to 10 for $N_{M}$, 10 to 150 for $N_{Q}$, 0.2 to 3.0 for λ, and 0.01 to 2.3 for γ. Experimental results with the combinations of these parameter values are analyzed below.

#### 4.7.1. Stationary Case

**Figure 10:**
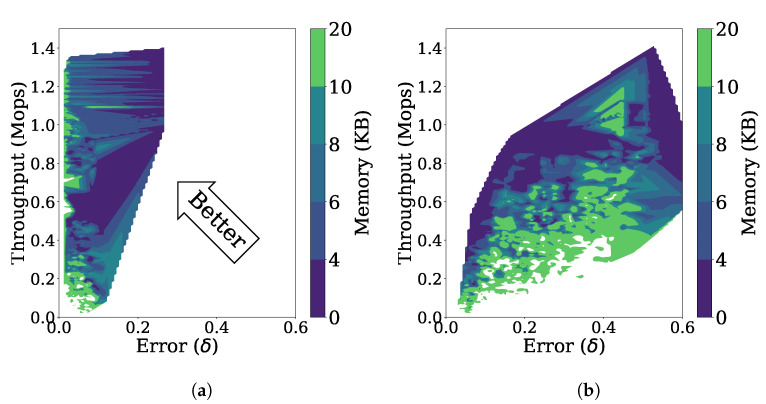
Performance of FlexSketch for various combinations of parameters’ values. (**a**) Stationary data stream. (**b**) Non-stationary data stream.

Figure 10a is a three-dimensional representation of the throughput, error, and memory usage for the stationary data stream as the parameters are changed. Many of the data points are located in the upper left side, indicating that FlexSketch achieves high throughputs and low errors over various combinations of the parameter values.

**Figure 11:**
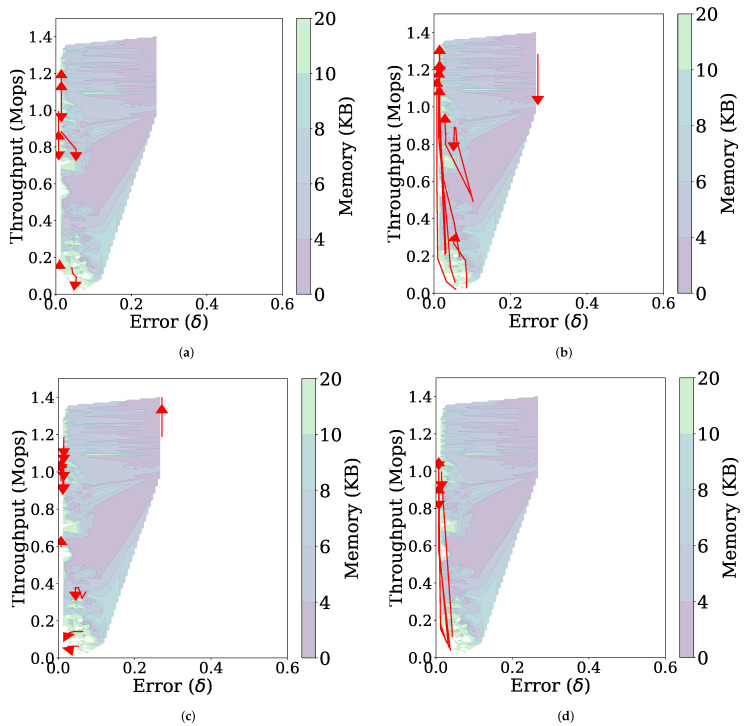
Trajectories (represented by red arrows) of the performance of FlexSketch for the stationary data stream as the value of each parameter increases (**a**) $N_{M}$ (**b**) $N_{Q}$ (**c**) γ (**d**) λ.

Figure 11 presents the effect of each parameter separately by increasing one of the four parameters while the others remain fixed. The following observations can be made. First, as $N_{M}$ increases, the throughput tends to decrease because of increased computational complexity for more histograms, while the error does not change (Figure 11a). Second, increasing $N_{Q}$ and γ results in decreased errors and increased throughputs in Figure 11b,d, respectively. The improved throughput is because using a larger *Q*, or increasing the threshold γ allows the computationally intensive MajorUpdate operation to be performed less frequently. Since the MajorUpdate operation adds a model representing the latest data to the data structure, performing less MajorUpdate operations reduces the dependence on the latest data, which improves the accuracy for the stationary data stream. Third, the value of λ does not affect much on the performance (Figure 11c).

#### 4.7.2. Non-Stationary Case

Figure 10b shows the throughput, error, and memory usage for the non-stationary data stream with incremental concept drift as the parameters are changed. Depending on the values of the parameters, the performance of FlexSketch may become degraded (i.e., larger errors, lower throughput, or larger memory consumption).

**Figure 12:**
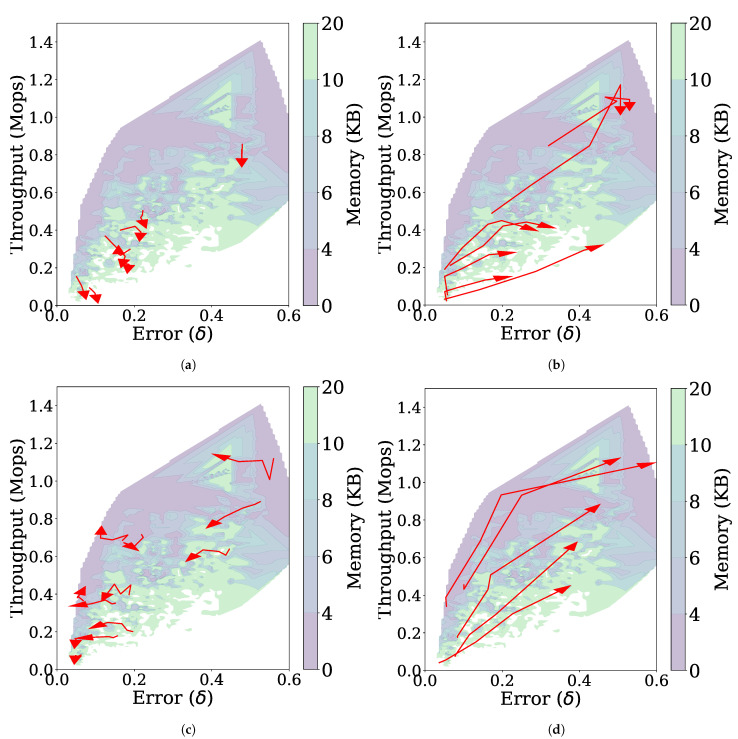
Trajectories (represented by red arrows) of the performance of FlexSketch for the non-stationary data stream with incremental concept drift as the value of each parameter increases. (**a**) $N_{M}$ (**b**) $N_{Q}$ (**c**) γ (**d**) λ.

Figure 12 shows how each parameter affects to the performance, from which we draw the following observations. First, as shown in Figure 12a, increasing $N_{M}$ results in lowering the throughput without changing the error much, which is due to the increased number of histograms as in the case of the stationary data stream. Second, when $N_{Q}$ or γ increases, a trade-off relationship is observed, i.e., the throughput increases but the error also increases (Figure 12b,d). Suppressing the MajorUpdate operation with increased $N_{Q}$ or γ improves the throughput, but prevents FlexSketch from accurately adapting to the concept drift. Third, by increasing λ, the contribution of past data is reduced and thus the error can be reduced.

## 5. Conclusions

In this paper, we have proposed the FlexSketch framework, which is an online algorithm based on an ensemble of histograms and consists of three operations: MinorUpdate, MajorUpdate, and Diagnose. Since it dynamically determines when to forget old data by observing divergence, it estimates probability distributions stably for stationary data streams without invoking the MajorUpdate operation. FlexSketch adapts to concept drift swiftly for non-stationary data streams by updating underlying model rapidly using MajorUpdate. As shown in Section 4.5.2, FlexSketch estimates probability distribution with high accuracy for data streams with sudden and incremental concept drift. Because FlexSketch utilizse simple histogram as the elemental data structure, it achieves high throughput update and query operations using only limited memory. The experimental results demonstrated the advantages of the method we propose in this paper. FlexSketch exhibits significantly improved speed compared to its alternatives. Moreover, FlexSketch adapts well to various non-stationary data streams while maintaining stability over temporal fluctuations. Nevertheless, FlexSketch has a disadvantage since it has multiple parameters. As discussed in Section 4.7, FlexSketch exposes some changes in throughput and accuracy according to parameters, which could be a burden of design choice in domain specific applications. While FlexSketch exhibits preferable characteristics, it needs to be extended to overcome the current limitation of supporting one-dimensional data only, which could be a drawback for some applications. In our future work, we plan to extend our method for multi-dimensional data streams. Because histogram is a simple and efficient underlying data structure for ensemble methods as shown in this paper, we will try to incorporate multi-dimensional histogram [58,59] to accommodate multi-dimensional data. In addition, we will explore applications that utilize probability estimation as a core building block. Drifting data stream classification [35] and anomaly detection in non-stationary data stream [60] would be good candidates to deploy FlexSketch for practical applications.

## Figures and Tables

**Figure 4:**
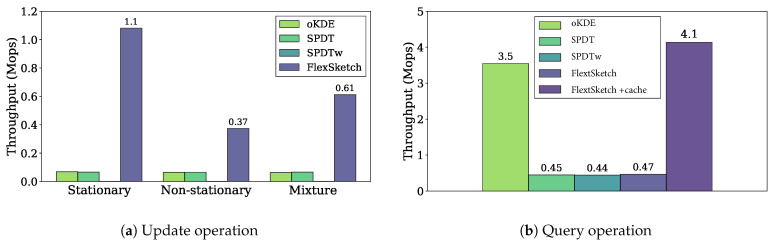
Throughput performance of the update and query operation (a higher value is better).
